# A Theoretical Framework for the Development of Views of One’s Own Aging

**DOI:** 10.1093/geroni/igaa057.017

**Published:** 2020-12-16

**Authors:** Sarah Marrs, Jennifer Inker, Madeline McIntyre, Leland Waters, Tracey Gendron

**Affiliations:** Virginia Commonwealth University, Richmond, Virginia, United States

## Abstract

Senior mentoring programs have been established that provide medical students exposure to a community-dwelling older adult mentor. The goal of these programs is to expose students to healthy older adults, increase knowledge of geriatrics, and prepare them to care for an aging population. However, even while participating in a senior mentoring program, health professions students still demonstrate some discriminatory language towards older adults (e.g., Gendron, Inker, & Welleford, 2018). In fact, research suggests ageist practices occur, intentionally or not, among all health professions and within assisted living and long-term care facilities (e.g., Bowling, 1999; Dobbs et al., 2008; Kane & Kane, 2005). There is reason to believe that how we feel about other older adults is a reflection of how we feel about ourselves as aging individuals. As part of an evaluation of a Senior Mentoring program, we found that students’ attitudes towards older adults were not significantly improved (t (92) = .38, p = .70). To further explore this, we collected subsequent qualitative data. Specifically, we asked students to respond to the open-ended prompt before and after completing their senior mentoring program: How do you feel about your own aging? Our findings have revealed just how complex students’ views towards aging and elderhood are, pointing to a need to develop a theoretical framework for how these views are formed. Thus, the results of this qualitative grounded theory study illustrate the stages of development medical students’ progress through as they come to accept themselves as aging humans.

